# Oesophageal carcinoma: comparison of *ex vivo* high-resolution 3.0 T MR imaging with histopathological findings

**DOI:** 10.1038/srep35109

**Published:** 2016-10-11

**Authors:** Yi Wei, Sen Wu, Dapeng Shi, Shewei Dou, Tingyi Sun, Peigang Ning, Cuihua Zhao, Ziyuan Li, Xiaodong Li, Feifei Gao, Linlin Li, Dandan Zheng, Shaocheng Zhu

**Affiliations:** 1 Department of Radiology, Zhengzhou University People’s Hospital, Zhengzhou 450003, China; 2 Department of Radiology, Henan Provincial People’s Hospital, Zhengzhou 450003, China; 3 Center of Thoracic Tumor, Zhengzhou University People’s Hospital, Zhengzhou 450003, China; 4 Center of Thoracic Tumor, Henan Provincial People’s Hospital, Zhengzhou 450003, China; 5 Department of Pathology, Zhengzhou University People’s Hospital, Zhengzhou 450003, China; 6 Department of Pathology, Henan Provincial People’s Hospital, Zhengzhou 450003, China; 7 GE Healthcare China, Beijing 100176, China

## Abstract

High-resolution magnetic resonance (MR) images clearly depict the normal oesophageal wall as consisting of eight layers, which correlates well with histopathological findings. In 56 (91.8%) of 61 lesions, the depth of oesophageal wall invasion determined through MR imaging was consistent with histopathological staging (r = 0.975, P < 0.001). The sensitivity, specificity and accuracy for the mucosa were 71.4%, 98.1%, and 95.1%, respectively, and the corresponding values for the submucosa were 82.4%, 95.5%, and 91.8%; for the muscularis propria, the sensitivity, specificity and accuracy were 100%, 95.7%, and 96.7%, respectively, and for the adventitia, these values were 100%, 100%, and 100%. The Cohen k values for interobserver agreement were excellent: K = 0.839, P < 0.001 (observer 1 vs. observer 2); K = 0.908, P < 0.001 (observer 1 vs. observer 3); and K = 0.885, P < 0.01 (observer 2 vs. observer 3). High-resolution *ex vivo* MR images obtained with a 3.0 T scanner can be used to precisely evaluate oesophageal carcinoma invasion and provide good diagnostic sensitivity, specificity and accuracy.

Oesophageal carcinoma is the eighth most common cancer worldwide, and its incidence is increasing^1^. However, the overall prognosis of oesophageal carcinoma remains poor due to the late stage and the poor preoperative stage at diagnosis^2^,^3^,^4^. Accurate preoperative staging of oesophageal carcinoma has been extremely difficult using routine imaging modalities^5^,^6^,^7^. Computed tomography (CT) is widely employed to identify the locations and relationships of surrounding organs and to assess distant metastasis of oesophageal carcinoma, but it does not reveal the invasion of the specific layer of the oesophageal wall due to poor soft-tissue contrast^8^,^9^. Endoscopic ultrasonography (EUS) can be used to depict the oesophageal layers but entails many inherent problems, including high operator dependency and limitations in cases of a stenotic oesophageal wall; the fact that EUS is a somewhat invasive procedure also restricts the clinical application of this technique^10^,^11^.

Magnetic resonance imaging (MRI) has been employed to evaluate oesophageal wall invasion by oesophageal carcinoma *in vivo*, and its potential as an alternative imaging modality to CT and EUS has been demonstrated^12^,^13^,^14^,^15^,^16^. High-resolution T2-weighted images obtained using a 1.5 T MRI system depict three different layers of the normal oesophageal wall^12^. PET/MR imaging exhibits acceptable accuracy for T and N staging for oesophageal carcinoma^13^. However, despite the satisfactory stage accuracy, the signal characteristics of the precise histopathological layers still cannot be obtained through *in vivo* MR imaging. Ultra-high-field 4.7 T and 7.0 T MR imaging^17^,^18^ of *ex vivo* oesophageal specimens can achieve an ultra-high spatial resolution and significantly improves the ability to observe the precise anatomical layers; however, ultra-high-field MR scanners are seldom used in clinical practice. Moreover, oesophageal specimens are generally formalin fixed, which does not allow the natural signal intensity of the oesophageal wall to be detected. To our knowledge, there have been no reports on the application of a 3.0 T MR system to depict the signal characteristics of precise oesophageal layers without formalin fixation and to assess oesophageal wall invasion in corresponding histopathological slices.

The purpose of this study is to prospectively determine the feasibility of 3.0 T MR imaging for evaluating the oesophageal layers of the normal, unfixed oesophageal wall *ex vivo* and to subsequently assess the depth of oesophageal wall invasion by oesophageal carcinoma.

## Results

### Signal intensity characteristics of the normal oesophageal wall

**Figure 1:**
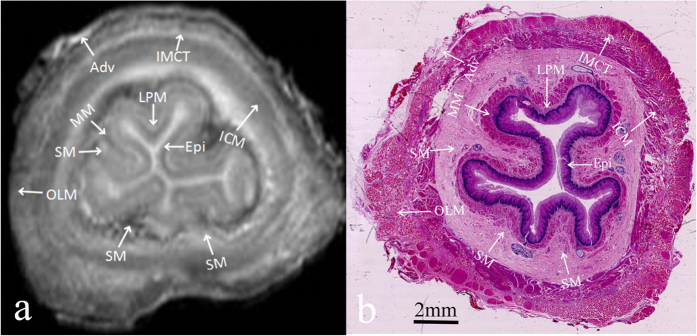
Normal oesophageal wall. (**a**) Transverse *ex vivo* T2-weighted image depicting the normal oesophageal wall as having 8 layers (white arrow): epithelium (Epi; hypointense), lamina propria mucosae (LPM; iso- to hyperintense), muscularis mucosae (MM; iso- to hypointense), submucosa (SM; mixed intensity), inner circular muscle (ICM; isointense), intermuscular connective tissue (IMCT; iso-to hyperintense), outer longitudinal muscle (OLM; hypointense), and adventitia (Adv; hyperintense). (**b**) Transverse histologic section of the normal oesophageal wall depicting the epithelium, lamina propria mucosae, muscularis mucosae, submucosa, inner circular muscle, intermuscular connective tissue, outer longitudinal muscle, and adventitia (hematoxylin-eosin stain; original magnification, ×10).

The signal intensity characteristics of the normal oesophageal wall under high-resolution T1-weighted imaging (T1WI) and T2-weighted imaging (T2WI) are shown in Table 1. There was no disagreement about the signal intensity between the three observers in any of the cases. In T1-weighted images, the oesophageal layers were similarly isointense, and these images could therefore not be used to determine or observe the individual oesophageal layers. In high-resolution T2-weighted MR images, the mucosa was observed as three layers (Fig. 1a). The epithelium was hypointense, while the muscularis mucosae was iso- to hypointense, and these two layers were separated from the iso- to hyperintense lamina propria mucosae. In high-resolution T2-weighted MR images, the submucosa exhibited a mixed signal intensity, and the histopathological findings demonstrated that it contained loose connective tissue, capillary vessels, and lymph channels. The muscularis propria was also subdivided into three layers. The inner circular muscle was isointense, while the intermuscular connective tissue was iso- to hyperintense, and the outer longitudinal muscle was hypointense. The adventitia was hyperintense in T2-weighted MR images. Thus, high-resolution T2-weighted MR images clearly depicted the normal oesophageal wall as consisting of eight layers, which corresponded well to histopathological findings (Fig. 1b).

### Evaluation of carcinoma invasion *ex vivo*

**Figure 2:**
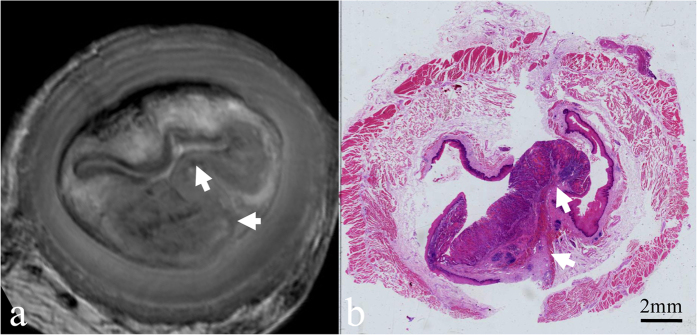
Oesophageal carcinoma that has invaded the mucosa. (**a**) Transverse *ex vivo* T2-weighted image demonstrating that the carcinoma has invaded into the mucosa (white arrow), and the iso- to hypointense muscularis mucosae is still intact (white arrow). (**b**) Transverse histologic section showing that the muscularis mucosae is intact (white arrow) (hematoxylin-eosin stain; original magnification, ×10).

**Figure 3:**
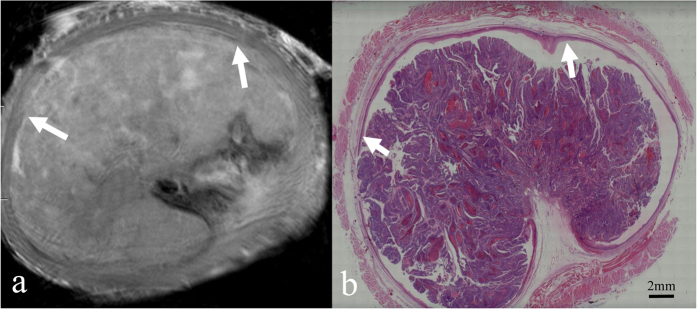
Oesophageal carcinoma invading the submucosa. (**a**) Transverse *ex vivo* T2-weighted image demonstrating that the carcinoma has invaded into the submucosa (white arrow), and the inner circular muscle is still intact. (**b**) Transverse histologic section showing that the tumour has invaded the submucosa (white arrow) (hematoxylin-eosin stain; original magnification, ×10).

**Figure 4:**
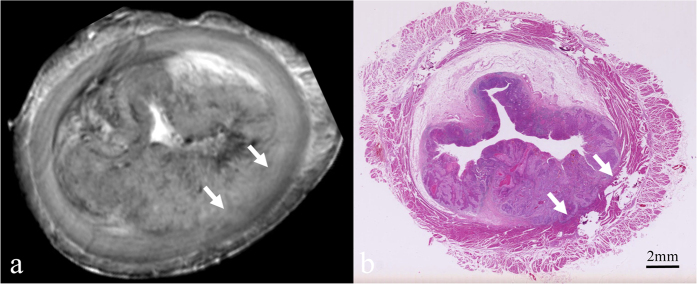
Oesophageal carcinoma invading the muscularis propria. (**a**) Transverse *ex vivo* T2-weighted image showing that the slightly higher signal intensity carcinoma has compressed the inner circular muscle (white arrow) without clear delineation, and the inner circular muscle is obviously thinner. (**b**) Corresponding histologic section showing carcinoma invading the inner circular muscle (white arrow), and the submucosa has been infiltrated. (hematoxylin-eosin stain; original magnification, ×10).

**Figure 5:**
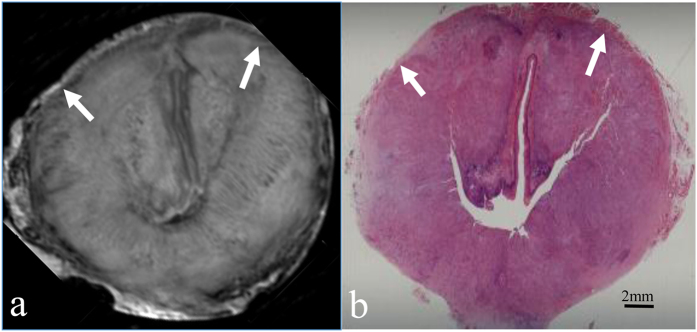
Oesophageal carcinoma that has invaded the adventitia. (**a**) Transverse *ex vivo* T2-weighted image showing that the tumour has invaded the adventitia (white arrow). (**b**) Transverse histologic section showing that the tumour has invaded the adventitia (white arrow). (hematoxylin-eosin stain; original magnification, ×10).

According to histopathological analysis, the 61 oesophageal carcinomas in our series consisted of 7 carcinomas confined to the mucosa (Fig. 2a,b), 17 that had invaded the submucosa (Fig. 3a,b), 14 that had infiltrated the muscularis propria (Fig. 4a,b), and 23 that had penetrated the muscularis propria and ex- tended into the adventitia (Fig. 5a,b). The depth of carcinoma invasion into the oesophageal wall was clearly demonstrated under T2WI, but T1WI was not useful for diagnostic evaluation. According to the histopathological stages of the 61 oesophageal specimens, high-resolution *ex vivo* MRI was used to correctly stage 56 (91.8%) of 61 oesophageal carcinomas, with 5 cases of incorrect diagnoses. Among these 5 cases, 2 cases confined to the mucosa appeared to exhibit submucosal invasion; and 2 cases confined to the submucosa were defined as muscularis propria invasion, as the submucosa and muscularis were obviously compressed. Therefore, based on the MR images, the severity of these cases was overestimated. In contrast, 1 case that involved invasion into the submucosa was diagnosed as mucosal invasion because the submucosal invasion was so microscopic that it was not observable due to the MR resolution. Detailed information about the stage of oesophageal layer invasion in the high-resolution *ex vivo* MR imaging results compared with histopathological findings is listed in Table 2.

### Diagnostic accuracy of MR imaging

Table 3 shows the diagnostic performance of high-resolution *ex vivo* MR imaging in the evaluation of carcinoma invasion of the oesophageal wall. In 5 (8.2%) of 61 lesions, the depth of carcinoma invasion into the oesophageal wall based on the MR images was not consistent with the histopathological findings; a Spearman correlation coefficient of 0.975 was obtained between the results of MR imaging and the histologic staging findings (P < 0.001), indicating that the correspondence was excellent. Furthermore, for 7 (11.5%) of 61 lesions, stage determination was not consistent between observer 1 and observer 2 (K = 0.839, P < 0.001); in 4 (6.6%) of 61 lesions, stage determination was not consistent between observer 1 and observer 3 (K = 0.908, P < 0.001); and in 5 (8.2%) of 61 lesions, stage determination was not consistent between observer 2 and observer 3 (K = 0.885, P < 0.001).

## Discussion

Accurate preoperative staging of oesophageal carcinoma has always been limited because the available imaging modalities recommended by the NCCN Clinical Practice Guidelines for oncology cannot be used to accurately evaluate carcinoma invasion of the oesophageal wall^19^. However, compared with the AJCC^6th^ guidelines for the staging of oesophageal carcinoma, the most recent AJCC^7th^ staging guidelines pay more attention to the depth of carcinoma invasion^20^. Regarding T staging, the previous T1 was divided into T1a and T1b, while T4 was divided into T4a and T4b, and different stages determine different therapeutic strategies. For example, for T1 tumours, endoscopic resection (ER) followed by ablation is the primary treatment option for T1a, whereas oesophagectomy is the primary treatment option for T1b; therefore, the new staging classification has made the limitations of the available imaging modalities even more obvious^21^.

Compared with CT and EUS, MRI provides high-resolution images of soft tissue structures, which enable more accurate differentiation of oesophageal layers compared with CT and EUS^12^,^22^,^23^,^24^. In this study, high-resolution T2-weighted MR images clearly depicted the oesophageal wall as consisting of 8 layers, which correlated well with the histopathological findings. Histologically, the epithelium layer consists of stratified squamous cells lining the surface of the mucosa with a higher cellular density and less interstitial fluid; therefore, the epithelium layer appears isointense in MR images. The lamina propria consists of dense connective tissue with capillaries and lymph vessels; therefore, in contrast to the isointense epithelium, the lamina propria appears to be of mixed intensity. Thus, the signal intensity of each layer depicted in MR images reflects the inner histologic structure and cellularity levels.

In the current study, T2-weighted MR images were found to be most useful for assessing tumour invasion into the oesophageal wall. The normal oesophageal layers and the carcinomas appeared isointense in T1-weighted images, with poor soft-tissue contrast, and these images therefore cannot be used to assess carcinoma invasion. The optimal sequence demonstrated in this study was consistent with the results obtained by Riddle^22^ and Yamada^15^,^17^. In T2-weighted images, carcinomas confined to the mucosa (T1a) exhibited a slightly higher signal intensity that had invaded mucosal layer, but the muscularis mucosae was found to be intact. Carcinomas invading the submucosa (T1b) appeared to be irregular solid masses with a slightly higher signal intensity, in contrast to the mixed signal intensity of the submucosa. However, the isointense signal of the inner circular muscle was found to be intact. Carcinomas infiltrating the muscularis propria (T2) partially replaced that layer, and the inner circular muscle or outer longitudinal muscle was involved. T2-weighted images showing a carcinoma extending to the adventitia (T3 and T4) demonstrated that the muscularis propria was completely disrupted by the carcinoma, and the high signal intensity of the adventitia was affected.

Our data demonstrated that high-resolution T2-weighted MR images enabled precise evaluation of carcinoma invasion into the oesophageal wall for 56 (91.8%) of 61 lesions. The overall stage accuracy in this study was consistent with a previous study performed by Yamada using 4.7 T and 7.0 T MRI 17,18. However, compared with this previous study, the experimental specimens used in the present study were not formalin fixed. Imai *et al*.^25^ found that in T2-weighted MR images, the signal intensity of the muscularis propria of colorectal wall specimens was somewhat higher in fresh specimens than in fixed specimens. Kanawaku *et al*.^26^ reported that the contrast of brain tissue was qualitatively reduced in fixed brain specimens compared with unfixed brains. Thus, fresh oesophageal specimens may be helpful for more effectively determining the MR signal intensity and show better contrast than fixed specimens. In addition, the imaging time was also significantly shorter in the present study compared with Yamada’s study^17^,^18^.

*In vivo* MR imaging is used for the staging of oesophageal carcinoma. In a study on oesophageal carcinoma, Riddle *et al*.^12^,^22^ used an external surface coil and T2-weighted MR images to show three layers of the oesophageal wall. Lee *et al*.^13^ performed PET/MR imaging with a surface coil, and accurate tumour staging was possible in 10 (66.7%) of 15 patients. Despite the acceptable staging accuracy achieved through *in vivo* MR imaging of oesophageal carcinoma, more precise observations of histopathological layers are not currently possible, particularly with 3.0 T MR imaging. The results of this study deepen the understanding of the characteristics and appearances of the oesophageal wall layers in *ex vivo* MR images and lay the foundation for precise *in vivo* staging of oesophageal carcinoma using 3.0 T MRI.

We acknowledge some limitations of our study. First, corresponding *in vivo* MR imaging of the patients was not conducted, and the precise oesophageal layers observed in *ex vivo* MR images cannot be associated with *in vivo* MR images. Second, the N-stage of the carcinomas was not investigated. Because the N-stage is important for the selection of a therapeutic scheme and is heavily associated with the prognosis, the N-stage will be included in our future studies. Finally, the rather limited number of T1a samples calls the extent to which these findings are representative into question; therefore, we should continue to collect more T1a samples.

In conclusion, the results of the preliminary study demonstrated that high-resolution *ex vivo* 3.0 T MR imaging is able to clearly depict the precise histopathological layers of the oesophageal wall *ex vivo*. High-resolution MR images of these *ex vivo* specimens provided excellent diagnostic accuracy for assessing oesophageal carcinoma invasion.

## Materials and Methods

### Patients

This study was approved by the institutional review board (Zhengzhou University People’s Hospital), and written informed consent was obtained from all patients. This study was conducted in accordance with the Declaration of Helsinki. Between July 2014 and March 2016, 85 consecutive patients with suspected oesophageal carcinoma lesions underwent gastroscopy biopsy. Among these patients, 24 subjects were excluded for the following reasons: 1, histopathological findings were not available because operations were not performed (n = 8); 2, patients underwent oesophagectomy after preoperative chemoradiotherapy (n = 6); 3, the oesophageal lesions were determined to be benign tumours based on histopathological evaluation (n = 6); and 4, the image quality was unsatisfactory (n = 4). The final study population consisted of 61 oesophageal specimens from 61 consecutive patients (39 men and 22 women; mean age, 64.70 ± 7.97 years; range, 40–84 years) with newly diagnosed oesophageal carcinoma. The length of the oesophageal specimens ranged from 40 to 250 mm (165 ± 40 mm, mean ± std). Histologically, there were 57 oesophageal squamous carcinomas and 4 oesophageal adenocarcinomas. In addition, 9 lesions were located in the upper oesophagus; 43 were located in the middle oesophagus; and 9 were located in the lower oesophagus.

### Imaging technique

MRI exams were performed on fresh, unfixed oesophageal specimens within 10-15 minutes after the resection. For all examinations, analyses were carried out using a 3.0 T MR system (Discovery MR750, GE Healthcare, Milwaukee, USA). A four-channel phased-array 3.5-inch animal coil was used for all measurements. The orientation of the T1WI and T2WI sequences was set to transverse. The maximum transverse diameter of the tumour was marked with a string before MR imaging, and the specimen was placed directly on the surface coil with the marked area as the scanning centre. The interspace between the coil and the resected oesophagus was filled with sterile gauze to reduce vibration artefacts. High-resolution T1WI sequence images were acquired using a spin-echo sequence with the following parameters: repetition time/echo time, 588/68.0 ms (effective); slice thickness, 3.0 mm with no gap; field of view, 60 × 36 mm^2^; matrix size, 256 × 192; and NEX, 2. High-resolution T2WI sequence images were acquired using an FRFSE (fast recovery fast spin-echo) sequence, and the imaging parameters were as follows: repetition time/echo time, 5500/85 ms (effective); slice thickness, 3.0 mm with no gap; field of view, 60 × 36 mm^2^; echo train length, 14; matrix, 512 × 352; and NEX, 14. The total scanning time was approximately 27 minutes (27.59 ± 3.217, mean ± std).

### Histopathological examination

After MR imaging, the oesophageal specimens were fixed with formalin for 24 hours, and appropriately fixed specimens were subsequently sectioned transversely, so that the orientation between the MR plane and specimens could be matched. The specimens were embedded in paraffin and cut into 5-μm slices with a microtome, then stained with hematoxylin-eosin (H-E). Photographs of the histopathological slices were scanned with a pathological slice scanner (Motic pro 285A). An experienced gastrointestinal pathologist (T.S, with 19 years’ experience reading histopathological slices) who was blinded to the MR findings identified carcinoma invasion of the oesophageal wall.

### Imaging analysis

An independent blinded analysis of the MR images was performed by three experienced radiologists (S.Z, D.S, P.N, with 18, 21 and 15 years of experience reading MR images, respectively), who were blinded to the findings of the histopathological examinations. MR images were reviewed for the signal intensity and depth of mural invasion. According to the stages indicated by the seventh American Joint Committee on Cancer (AJCC^7th^) 27, the depth of carcinoma invasion into the oesophageal wall was classified according to the layer invaded (mucosa, submucosa, muscularis propria or adventitia). A fourth radiologist (S.D, with 12 years of experience reading MR images) who did not participate in the interpretation of the *ex vivo* MR images matched the MR images with magnified histopathological photographs. The MR images and histopathological slices were matched based on the depth of carcinoma invasion, tumour contours, adjacent lymph nodes and oesophageal lumen morphology.

### Statistical analysis

Numerical variance is indicated as the mean and standard deviation. Spearman correlation coefficient analysis was used to compare the T staging results based on MR imaging with those of the histopathological analysis. A P value of less than 0.05 was considered to indicate statistical significance. Interobserver agreement was compared using Cohen k values: a value of 0.4–0.6 indicates moderate agreement; 0.6–0.8 indicates substantial agreement; and a value greater than 0.8 indicates almost perfect agreement. In addition, the sensitivity (SE), specificity (SP), accuracy (AC), false positive rate (FPR), false negative rate (FNR), positive predictive value (PPV) and negative predictive value (NPV) were calculated. All statistical analyses were performed using a statistical software package (SPSS19.0 (SPSS Inc, Chicago, IL, USA)).

## Additional Information

**How to cite this article**: Wei, Y. *et al*. Oesophageal carcinoma: comparison of *ex vivo* high-resolution 3.0 T MR imaging with histopathological findings. *Sci. Rep.* **6**, 35109; doi: 10.1038/srep35109 (2016).

## Figures and Tables

**Table 1:** The Signal Intensity Characteristics of the Normal Oesophageal Layers in High-resolution T1- and T2- weighted MR Images.

Histopathological layers	Signal intensity characteristics of the oesophageal wall	
	T1-weighted MR images	T2-weighted MR images
Epithelium	isointense	hypointense
Lamina propria mucosae	isointense	iso- to hyperintense
Muscularis mucosae	isointense	iso- to hypointense
Submucosa	isointense	mixed-intense
Inner circular muscle	isointense	isointense
Intermuscular connective tissue	isointense	iso-to hyperintense
Outer longitudinal muscle	isointense	hypointense
Adventitia	isointense	hyperintense

Note: High-resolution T1-weighted MR images show the oesophageal layers as similarity isointense. Highresolution T2-weighted images depict the oesophageal wall as consisting of 8 layers, which corresponds well to histopathological findings.

**Table 2:** Comparison of High-resolution MR Images and Histopathological Findings for Evaluation of the Depth of Carcinoma Invasion.

MR findings	Histopathological findings			
	Mucosa (n = 7)	Submucosa (n = 17)	Muscularis propria (n = 14)	Adventitia (n = 23)
Mucosa	5	1	0	0
Submucosa	2	14	0	0
Muscularis propria	0	2	14	0
Adventitia	0	0	0	23

Note: The invasion of individual layers is based on the TNM classification of the American Joint Committee on Cancer (seventh edition)^27^.

**Table 3:** Diagnostic Accuracy of High-resolution Images for Evaluating Oesophageal Carcinoma Invasion.

Group	Mucosa (%)	Submucosa (%)	Muscularis Propria (%)	Adventitia (%)
SE	5/7 (71.4)	14/17 (82.4)	14/14 (100)	23/23 (100)
SP	53/54 (98.1)	42/44 (95.5)	45/47 (95.7)	38/38 (100)
AC	58/61 (95.1)	56/61 (91.8)	59/61 (96.7)	61/61 (100)
FPR	1/54 (1.9)	2/44 (4.5)	2/47 (4.3)	0/38 (0)
FNR	2/7 (28.6)	3/17 (17.6)	0/14 (0)	0/23 (0)
PPV	5/6 (83.3)	14/16 (87.5)	14/16 (87.5)	23/23 (100)
NPV	53/55 (96.4)	42/45 (93.3)	45/45 (100)	38/38 (100)

Note: SE: sensitivity; SP: specificity; AC: accuracy; FPR: false positive rate; FNR: false negative rate; PPV: positive predictive value; NPV: negative predictive value.
